# Complications of renal interventions: a pictorial review of CT findings

**DOI:** 10.1186/s13244-021-01048-9

**Published:** 2021-07-18

**Authors:** Jean S. Z. Lee, Jonathan Hall, Tom Sutherland

**Affiliations:** Medical Imaging Department, St Vincent’s Hospital Melbourne, 41 Victoria Parade, Fitzroy, VIC 3065 Australia

**Keywords:** Kidney, Surgery, Biopsy, CT, Complication

## Abstract

A number of potential vascular and non-vascular complications can arise from surgical, extracorporeal shock wave lithotripsy, radiotherapy and radiological renal interventions, including percutaneous image-guided biopsy and drainage. Computed tomography scan is usually one of the first and most important diagnostic imaging examinations requested when a potential complication is suspected. There are a wide range of common and uncommon potential complications from renal interventions. An understanding of underlying risk factors is important to reduce potential complications from renal intervention. Radiologists play a crucial role in recognising and diagnosing post-renal intervention complications on computed tomography scans, which could significantly improve the patient’s prognosis.

## Key points

- There are a wide range of complications from renal interventions.
- Knowledge of CT findings will enable the primary diagnosis of potential complications.
- An understanding of underlying risk factors may reduce complications from renal interventions.
- Early diagnosis of complications from renal intervention could improve the patient’s prognosis.

## Background

The incidence of common renal pathologies such as renal calculi and renal cell carcinoma has continuously increased over the past 50 years [1–4]. This has in part been attributed to the ubiquity of high-quality imaging such as ultrasonography, computed tomography (CT) and magnetic resonance imaging (MRI), leading to increased detection of smaller renal masses [5, 6] and of smaller renal calculi [2, 3].

The prevalence of renal calculi is estimated to be as high as 10–13% worldwide, increasing with age [2, 7]. Renal cell carcinoma makes up the majority (approximately 90%) of detected renal cancers [8] and has been increasing in incidence worldwide, with an age-standardised incidence rate of up to 16.7 per 100,000 [9].

The increased incidence of renal pathologies has significantly increased the number of renal interventions undertaken to diagnose and to treat renal pathologies. For example, the rates of intervention performed for urinary calculi have increased by approximately 17% in the past 20 years [10].

In addition, the increased detection of smaller and, often asymptomatic, renal pathologies have also led to the adoption of more conservative management options and to a progressive increase in a variety of more targeted and less invasive interventions [10, 11]. For example, a recent systematic review of data from six countries found that the use of extracorporeal shockwave lithotripsy and open surgery fell by 14.5% and 12%, respectively, whilst the use of ureteroscopy increased by more than 250% in the past two decades [10]. The use of nephron-sparing interventions such as partial nephrectomy and ablative techniques is also increasingly favoured over radical nephrectomies. An analysis of the National Cancer Database, recognised as the largest cancer registry in the world, showed that the proportion of patients receiving partial nephrectomy has almost doubled over approximately 10 years, from approximately 36.4% in 2004 to 61.2% in 2015 [12].

A wide range of interventions form part of the diagnostic and therapeutic pathway of renal diseases. These include renal procedures such as lithotripsy, radiological renal interventions such as image-guided core biopsies, as well as therapeutic interventions such as surgery, namely nephrectomy, and radiotherapy, whilst shown to have high rates of safety and efficacy, these interventions are also associated with some potential complications. The aim of this article is to familiarise the radiologist with the common and less common complications from various renal interventions. Risk factors that are more commonly associated with complications will also be summarised. This could help the radiologist to prevent, as well as to diagnose, complications from renal interventions.

## Surgical complications

### Nephrectomy and ablative therapy complications

Open and laparoscopic radical nephrectomy is the gold standard in the management of large renal masses [13]. For smaller lesions, nephron-sparing procedures such as partial nephrectomy or percutaneous therapies are increasingly favoured.

**Fig. 1:**
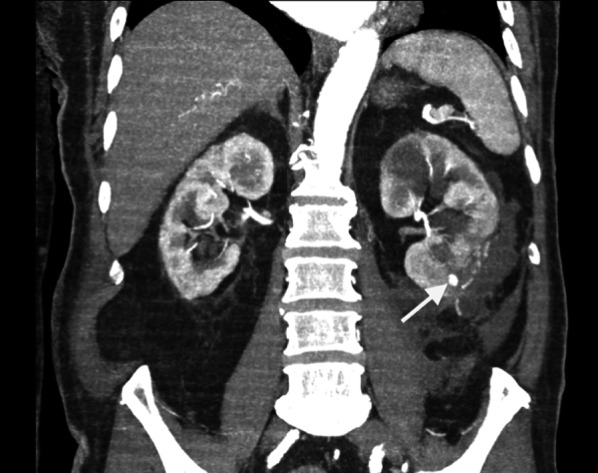
Arterial phase CT image of a pseudoaneurysm (white arrow) post-laparoscopic partial nephrectomy of the lower pole of the left kidney

**Fig. 2:**
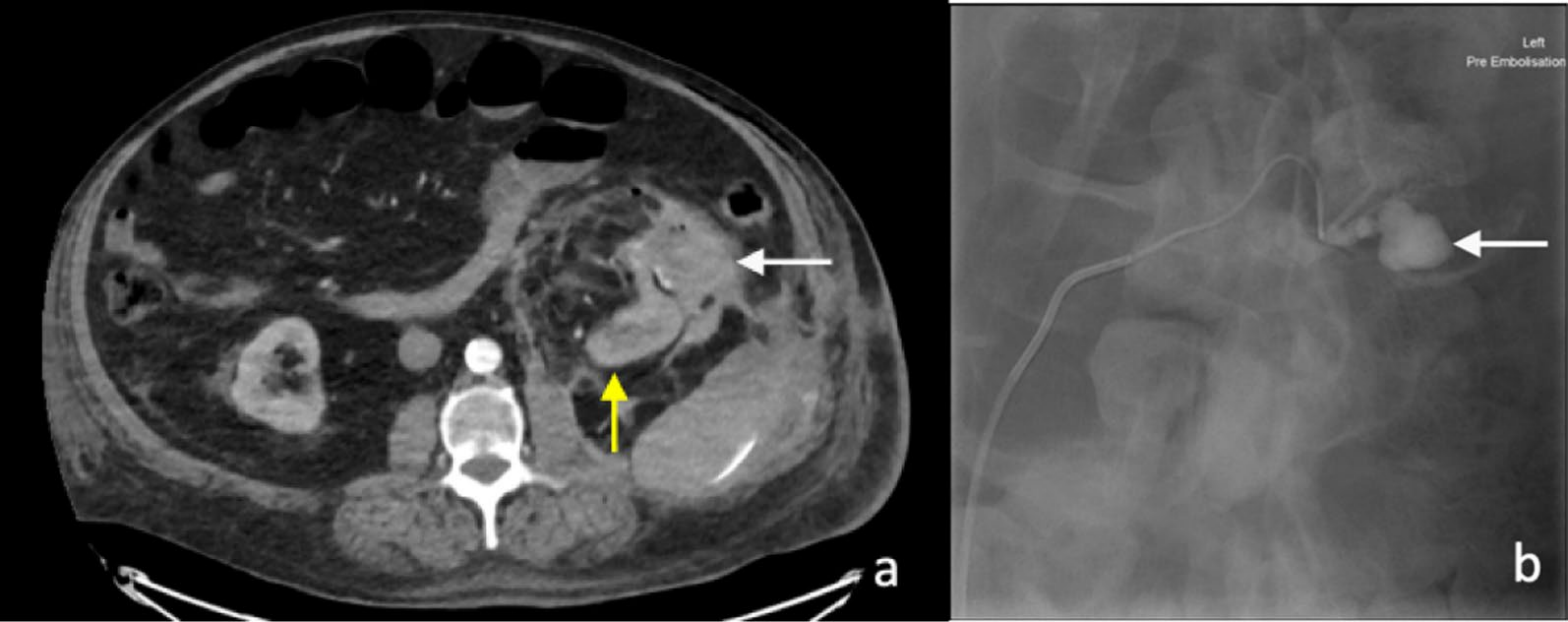
**a** Arterial phase CT image of perinephric haematoma (white arrow) at the site of partial nephrectomy. The residual left kidney is indicated by the yellow arrow. **b** Active extravasation of contrast (white arrow) was confirmed on DSA

Early CT imaging is indicated to assess the clinically suspected complications of surgery and to enable early management. Two main complications following nephrectomy requiring CT imaging is haemorrhage and urinary leakage [4]. Post-operative haemorrhage may arise from an unsecured artery, or days to weeks later due to the rupture of a pseudoaneurysm of an intrarenal artery (Fig. 1). The presence of a post-operative perinephric haematoma can be demonstrated by CT, ultrasound or MR imaging; however, the site of active haemorrhage is best demonstrated on a CT angiography (CTA), or ultimately, diagnostic angiography (DSA) (Fig. 2) [14]. A multiphase study is recommended, including non-contrast, arterial and subsequent portal venous phase approximately a minute after injection of a contrast bolus [15]. Acute haematomas are typically hyperattenuating (40–60 Hounsfield units) relative to the renal parenchyma on unenhanced CT images [16]. Small subcapsular haematomas appear crescenteric when small and biconvex when large on CT [16]. Extravasation of contrast material, indicating active bleeding, was first described by Sivit et al. [17]. The extravasated contrast has a density close to the density of either the aorta or other major adjacent arteries and is typically surrounded by lower attenuation haematoma [18]. The presence of vascular extravasation of contrast enables the identification of the anatomic site of injury to inform emergent treatment to prevent a potentially life-threatening haemorrhage [19].

A study of 1800 cases of open and laparoscopic partial nephrectomies found that approximately 5% of patients suffer significant blood loss requiring transfusion, with no significant difference in blood transfusion rates between the open or laparoscopic approach [20]. Asymptomatic pseudoaneurysms have been detected on CT scans in approximately 15% of patients following partial nephrectomy in the early post-operative period [21]. These usually spontaneously resolved, but a small number, approximately 1% in a case series, have required selective arterial embolisation [22].

**Fig. 3:**
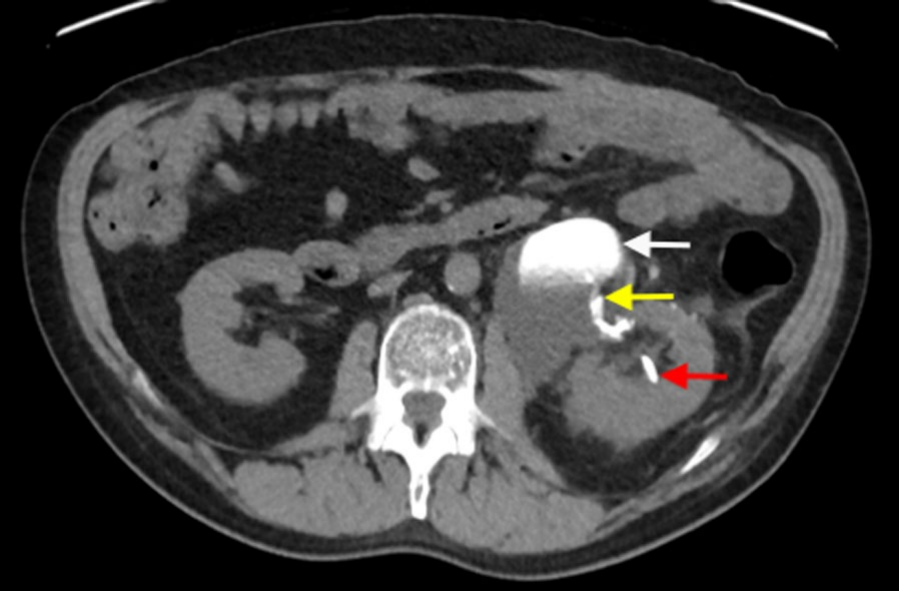
Delayed excretory phase CT image of contrast extravasation into a urinoma (white arrow) following left ureteric injury during a left partial nephrectomy. The yellow arrow demonstrates the site of active contrast extravasation from the left pelvi-ureteric junction, which was injured during the operation. A nephrostomy catheter (red arrow) was sited to decompress the collecting system and to facilitate urinary drainage. The patient developed persistent pelvi-ureteric junction obstruction and required a completion left nephrectomy

Urinary leakage has been reported in approximately 1% of patients following open or laparoscopic partial nephrectomy [22, 23]. It can occur from intra-operative injury to the renal pelvis, ureters or urinary bladder. This may be clinically suspected following flank pain, renal dysfunction or drainage of urine from a surgical drain. A urinoma may be detected as a perinephric collection on an ultrasound, CT or MRI scan, which may cause ureteric or vascular compression. The site of urinary leakage is most commonly demonstrated as contrast extravasation from the renal tracts or collecting system on a CT urogram study, performed approximately 10–15 min after intravenous administration of contrast (Fig. 3) [16].

**Fig. 4:**
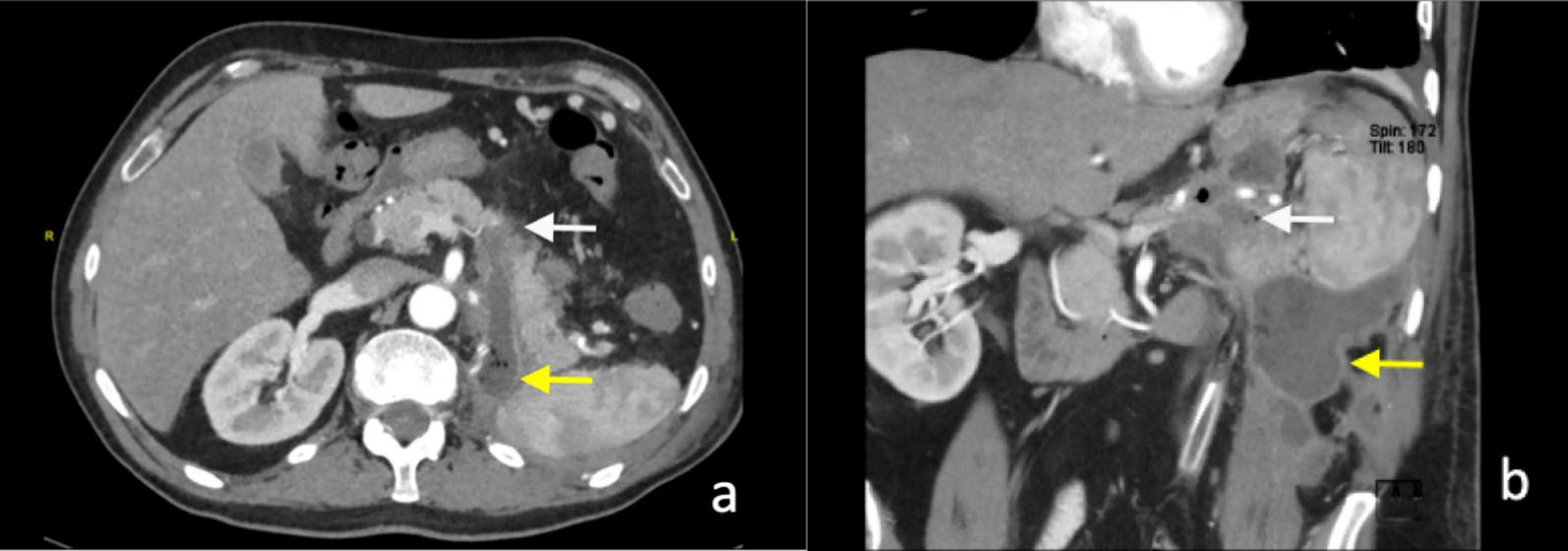
**a** Axial and (**b**) coronal images of pancreatic injury (white arrow) with a rim-enhancing fluid- and gas-containing collection (yellow arrow) following left nephrectomy for RCC

Intra-operative injuries to the adjacent structures can also occur post-renal surgery. Splenic injuries have been reported to occur in 4–13% of cases following left nephrectomy [24]. Pancreatic, liver and gastric injuries have also been reported following renal surgeries (Fig. 4) [25]. Bowel injury occurs in less than 1% of cases following laparoscopic surgery [26]. Rarely, pneumothoraces can be caused by diaphragmatic injury during dissection of the upper pole of the kidney.

Ischaemic injury can also occur following prolonged clamping intra-operatively or due to renal artery manipulation leading to thrombosis or stenosis. Renal tract obstruction could also occur following direct ureteric injury or ischaemia leading to ureteric stenosis [4]. This could lead to renal tract obstruction and subsequent urinary leakage.

### Urological complications following stone treatment

The surgical management of urinary tract stones has evolved from open surgery to a range of minimally invasive procedures. With the exception of complex staghorn calculi, a range of minimally invasive techniques have been employed by urologists to treat urinary tract calculi, including extracorporeal shock wave lithotripsy (ESWL), and ureteroscopy, flexible ureterorenoscopy and percutaneous nephrolithotomy (PCNL) [27]. The selection of each technique is influenced by the surgeon’s experience, the nature of the stone burden, stone location, anatomy of the urinary tract and patient preference.

**Fig. 5:**
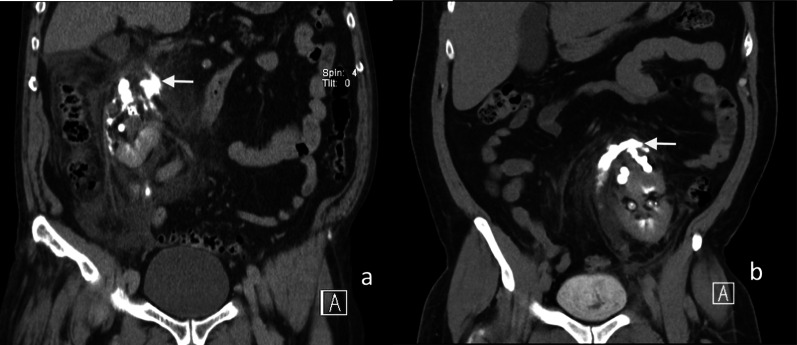
Coronal delayed excretory phase CT images of calyceal rupture and urinoma formation (white arrows) with perinephric extravasation of contrast in two patients. **a** Post-ESWL and (**b**) post-pyeloplasty

**Fig. 6:**
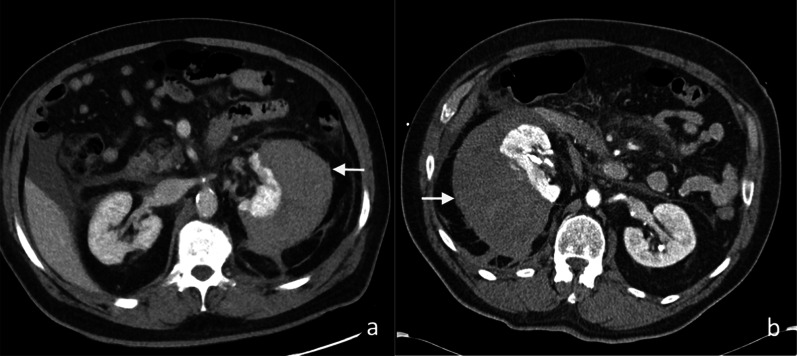
Axial contrast-enhanced CT images of (**a**) perinephric haematoma (white arrow), and (**b**) subcapsular haematoma (white arrow) post-ESWL in two patients

ESWL is a common minimally invasive treatment for renal tract calculi [28], with relatively few complications. One of the most common complications is the formation of a perinephric or subcapsular haematoma, with an incidence of approximately 4% (Fig. 5) [29]. The risk of a renal haematoma post-ESWL is significantly increased with increasing patient age [29], use of a therapeutic dose of low molecular weight heparin and the presence of an untreated urinary tract infection [30]. The resultant compression of the kidney from the perinephric or subcapsular haematoma has been reported to cause systemic hypertension, also known as Page kidney [31]. Repeated ESWL has been reported to cause ureteric perforation (Fig. 6), renal atrophy and irreversible damage to renal function [32, 33].

Ureteroscopy has also been shown to be effective in treating renal tract calculi with low complication rates [27]. The most common minor intra-operative complications were mucosal abrasions and bleeding, accounting for approximately 60% of the cases [34]. The incidence of serious complications such as bleeding and perforation is low at approximately 1–3% [34, 35]. Extra-ureteric stone migration and ureteric avulsion are also very rare (< 1%) [34, 35].

**Fig. 7:**
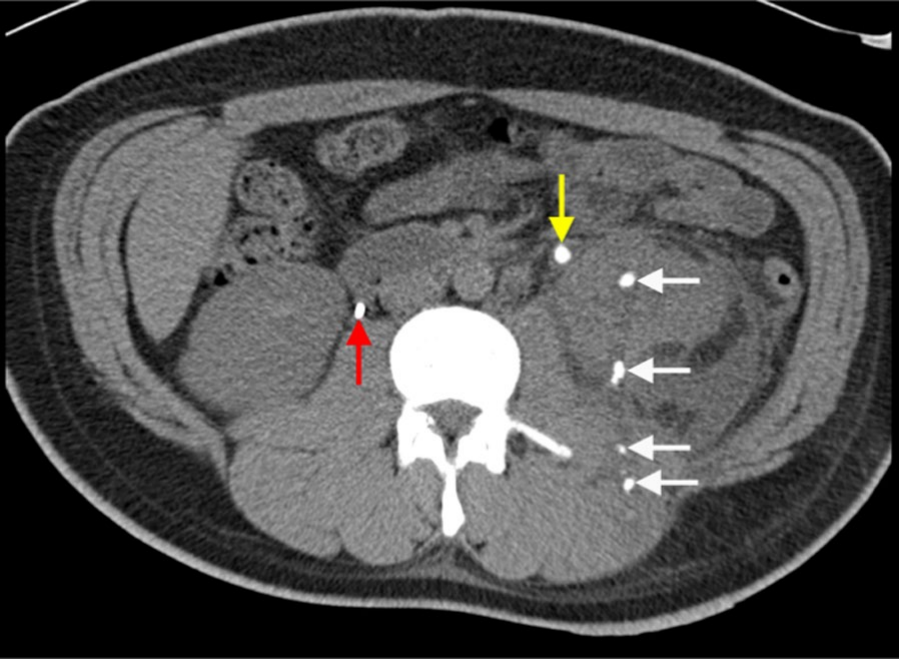
Unenhanced CT image of migration of renal calculi (white arrows) into the left percutaneous nephrolithotomy tract and a small left perinephric urinoma post-PCNL of a left staghorn calculus. There is a inferior migration of the ureteric calculus within the left proximal ureter (yellow arrow). A right-sided ureteric stent is in situ (red arrow)

PCNL has been shown to more effective in treating larger renal stones, with fewer retained stone fragments, but are associated with higher rates of complications such as fever, bleeding and renal scarring [36]. Post-procedural fever and bleeding have been reported to be as high as 10.5% and 7.8%, respectively [37]. Migration of residual stone fragments into the proximal ureter is rare and most fragments past spontaneously (Fig. 7) [38]. Injuries to the adjacent viscera such as bowel, liver, spleen and diaphragm are rare (< 1%) [39].

## Percutaneous complications

### Renal biopsy

More than half of renal cell carcinomas are now incidentally diagnosed [40]. Whilst the majority of incidentally detected renal masses are renal cell carcinomas, up to a fifth of incidentally detected renal masses are benign tumours [41]. There is a general consensus that the distinction of solid RCC from benign renal tumours such as renal oncocytoma and fat-poor angiomyolipomas, and of oncocytic tumours such as oncocytomas from chromophobe RCC, is not yet entirely possible or reproducible with imaging [42]. Despite advances in imaging techniques and algorithms, percutaneous image-guided renal biopsies are still needed to differentiate between benign and malignant renal tumours.

Current guidelines recommend targeted core biopsies of solid renal tumours to confirm or to exclude malignancy prior to treatment when the results may alter surgical management [43]. Core biopsy of cystic tumours, tumours originating in the collecting system or suspected urothelial cancer should not be performed [43]. Renal masses suspected of being haematologic, metastatic, inflammatory or infectious should also be biopsied to guide management, which is often very different from the RCC management pathway [44].

**Fig. 8:**
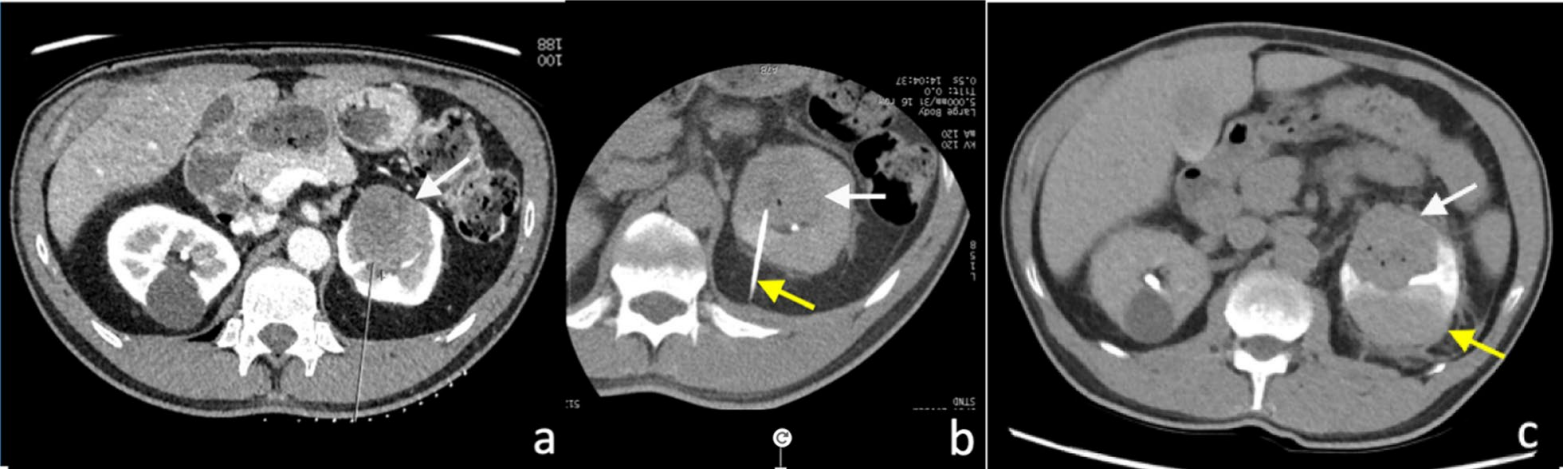
**a** Axial CT image pre-biopsy planning image of percutaneous posterior approach of a left renal cell carcinoma (white arrow) and (**b**) image demonstrating a coaxial biopsy needle system (yellow arrow) within the left renal cell carcinoma (white arrow). **c** Subcapsular haematoma (yellow arrow) post-percutaneous biopsy of the left anterior renal cell carcinoma (white arrow). The 18-gauge core biopsies confirmed the diagnosis of mucinous tubular and spindle cell renal cell carcinoma

In addition, percutaneous renal biopsy is essential in the diagnosis of intrinsic renal disease [45]. Indications vary between nephrologists. These include the diagnosis of idiopathic nephritic and nephrotic syndromes, the diagnosis of focal primary lesions, the detection of acute or chronic renal allograft rejection and the evaluation of antirejection therapy [46, 47]. Non-nephrotic proteinuria and isolated glomerular haematuria are usually regarded as conditions in which biopsy is not indicated [47]. Image-guided renal biopsies are usually performed with ultrasound or CT guidance (Fig. 8). The use of percutaneous image-guided renal biopsies could also reduce the number of nephrectomies performed for benign renal masses and for indolent renal carcinomas [12, 48, 49]. The pre-operative diagnosis of an indolent RCC could enable the clinical team to adopt a more conservative approach such as active surveillance, especially in older or frail patients. Patel et al. found a statistically significant correlation between the increased use of renal mass biopsy and the use of non-surgical management, including active surveillance [12].

It is, therefore, unsurprising that the use of percutaneous image-guided renal biopsies has been on the rise. In 2015, approximately 15.3% of patients presenting with a renal mass received a percutaneous biopsy, increased from approximately 8.0% in 2004–2007 [12]. There are concerns, however, that percutaneous image-guided renal biopsies remain underutilised in the management of renal masses [50] especially as up to 30% of surgically excised renal tumours were benign and small, measuring < 4 cm in size [51]. Approximately 32–56% of urologists surveyed indicated that they would not obtain pre-operative biopsy [50, 52]. The number of surgically resected benign renal masses in the USA increased by 82% from 2000 to 2009 [53]. In addition, the almost doubled incidence of RCC and the corresponding increased rates of nephrectomy over the past 20 years have not been matched by improved mortality rates for RCC [54]. On the contrary, the mortality rates for RCC have remained stable, suggesting overdiagnosis and overtreatment [54].

The diagnostic accuracy of 18-gauge core biopsy of renal masses is generally high, up to > 90% [55]. The non-diagnostic rate of core biopsy of renal masses is approximately 10–20% [43, 56]. The non-diagnostic rate is decreased by approximately 80% with a repeat biopsy [56, 57]. As such, core biopsies are favoured over fine needle aspirates in the diagnosis of solid renal tumours [58]. Core biopsy of renal tumours is highly sensitive (97.5%, CI 96.5–98.5) and specific (96.2%, CI 90.7–100) when diagnostic, reducing surgical excision for the majority of patients with a benign biopsy (approximately 80%) [56]. It has a low false-positive rate of 4.0%, but has a limited negative predictive value of approximately 63.3% (CI 52.4–74.2). 90% of the patients with a non-diagnostic result were found to have malignancy following surgical excision [56].

**Fig. 9:**
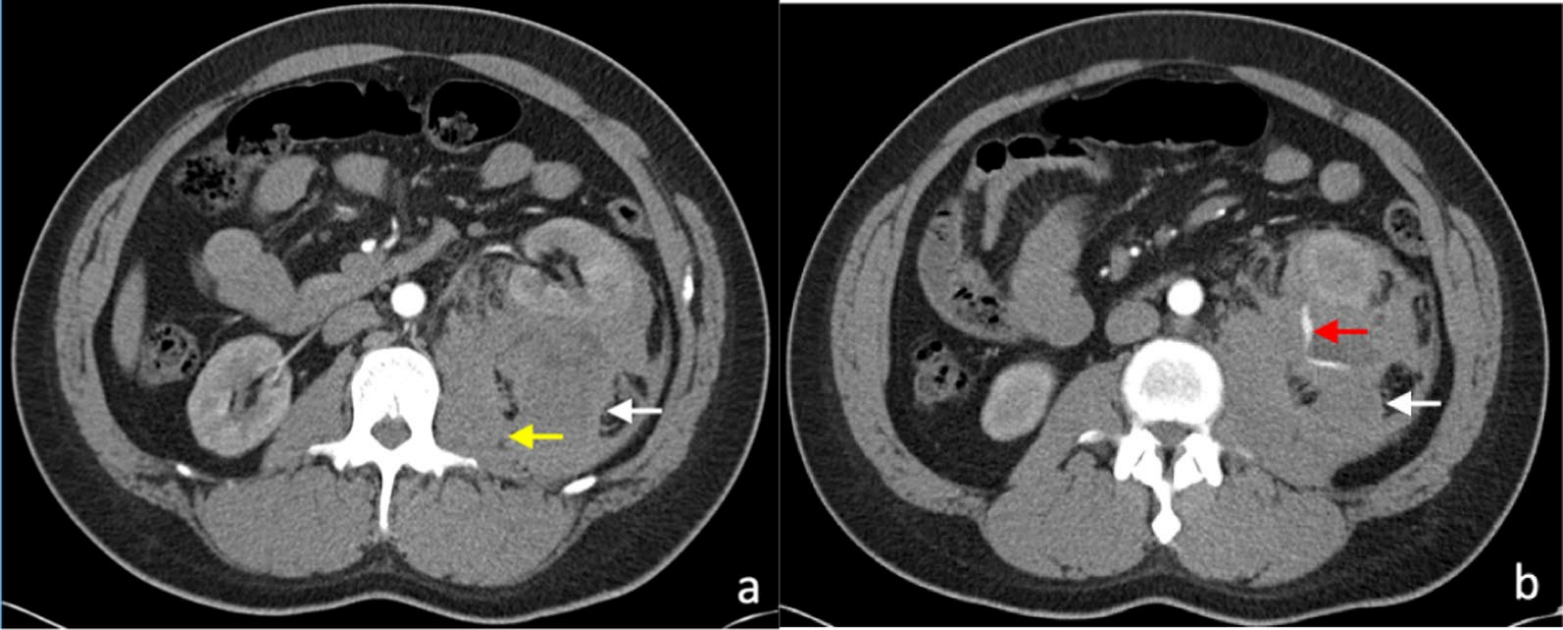
Post-contrast arterial phase axial CT images of a patient following two 14-gauge non-targeted core biopsies of the left kidney, demonstrating (**a**) left perinephric haematoma (white arrow). There is also a haematoma of the left psoas muscle, which is expanded (yellow arrow). **b** Active extravasation of contrast from a left inferior segmental renal artery (red arrow) was demonstrated inferiorly within the perinephric haematoma (white arrow). The active haemorrhage was successfully treated with embolisation coils of the left inferior segmental renal artery. The core biopsies confirmed IgA nephropathy

**Fig. 10:**
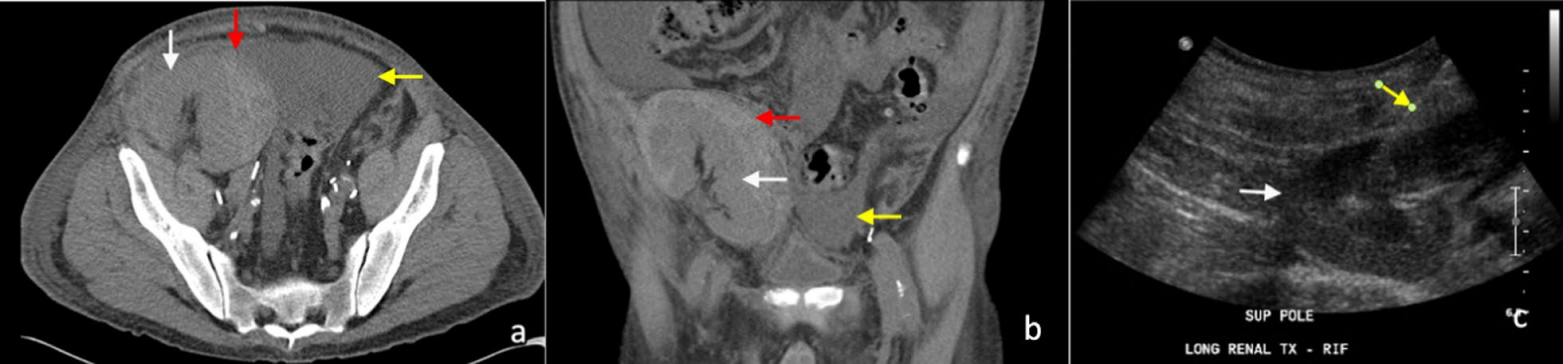
Unenhanced (**a**) axial and (**b**) coronal, CT images of a subcapsular haematoma (red arrow) following an ultrasound-guided non-targeted 14-gauge core biopsy of a right iliac fossa renal transplant allograft (white arrow). The patient had a moderate volume of ascites (yellow arrow) present prior to the biopsy. **c** An ultrasound-guided non-targeted core biopsy of the right iliac fossa renal graft (white arrow) was performed with a 14-gauge needle (yellow arrow) for deteriorating renal function. The single-pass 14-gauge core biopsy demonstrated features of acute on chronic graft rejection

Core biopsy of renal tumours is safe with low rates of serious complications [42, 58]. The median overall complication rate has been reported as approximately 8.1% (IQR 2.7–11.1%). Of this, the most commonly reported complication is minor haemorrhage or haematoma not requiring treatment [58], which is reported to be as high as 4.9% (Figs. 9, 10) [56]. The incidence of severe haemorrhage requiring treatment is very low at approximately 0.4% to 0.7% [56, 58]. Other less common complications include clinically significant pain (1.2%), gross haematuria (1.0%) and pneumothorax (0.6%) [56]. The risk of tumour seeding from RCC along the percutaneous biopsy tract is very low, estimated at approximately 0.01% [59], potentially owing to the slow-growing nature of RCC and the use of a coaxial biopsy technique [60].

**Fig. 11:**
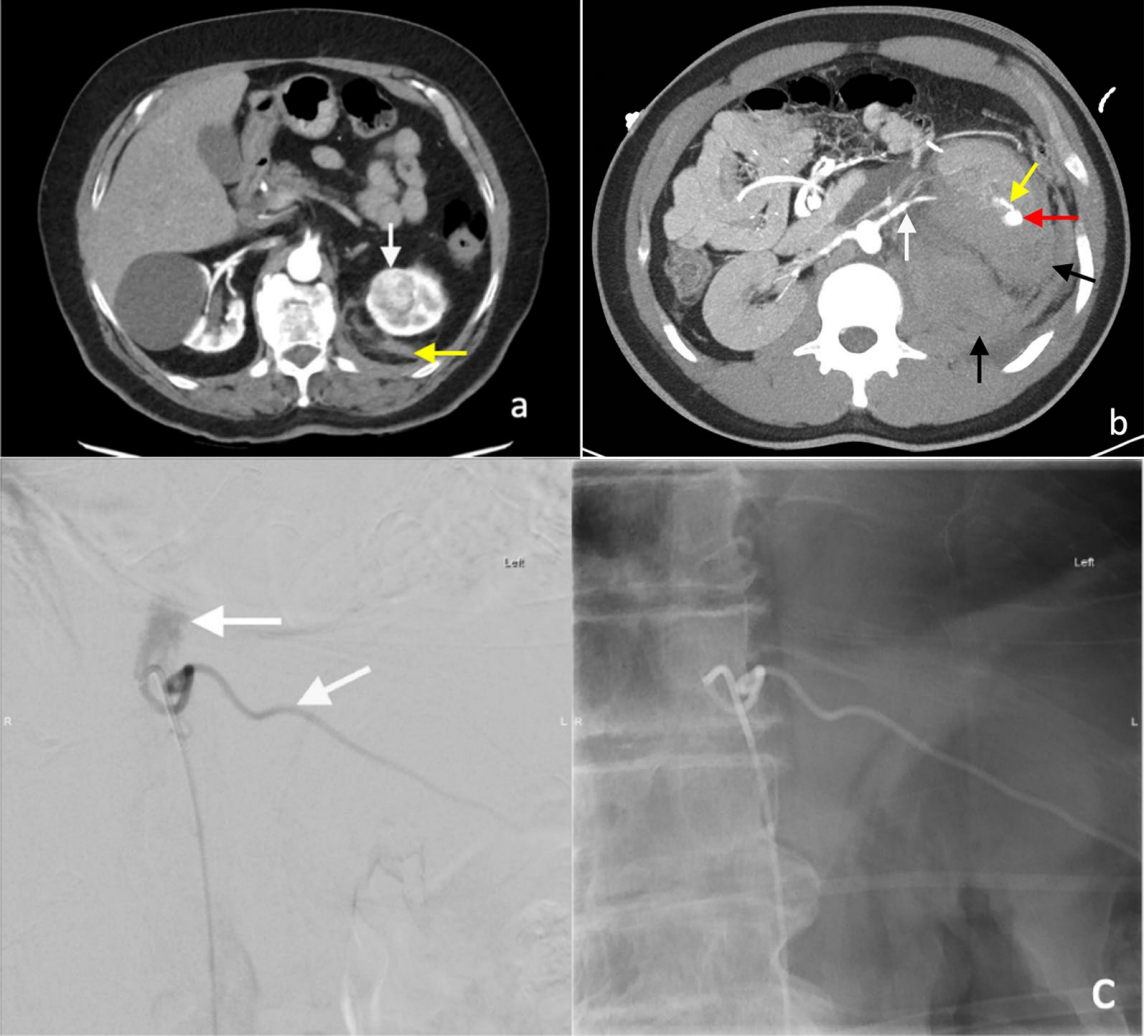
**a** Axial contrast-enhanced arterial phase CT image following a CT-guided 18-gauge core biopsy of a left upper pole renal tumour (white arrow) with adjacent perinephric haematoma and stranding (yellow arrow). **b** A more superior arterial phase axial CT image in the same patient demonstrated active extravasation of contrast (white arrow) within the small left haemothorax (yellow arrow) from a left intercostal artery at T11 (red arrow). **c** Active contrast extravasation (white arrow) from a left T11 intercostal artery (yellow arrow) was confirmed on angiography and successfully treated with embolisation coils

The incidence of pseudoaneurysm following percutaneous renal biopsy is unknown, probably because most are asymptomatic [60] and incidentally detected on surveillance imaging, including in our cases (Fig. 11b). These were successfully treated with embolisation. Maturen et al. reported a pseudoaneurysm as a late complication of renal biopsy, following delayed presentation of the patient 3 months later with retroperitoneal haemorrhage [60]. Pseudoaneurysms can be detected as a round or oval collection of extravascular arterial contrast that is surrounded by and contained in an adjacent haematoma. Active haemorrhage tends to track into surround tissues and has a linear or flame-like appearance (Figs. 9b, 11), whereas pseudoaneurysms have sharply defined edges and do not blend with the adjacent haematoma [16, 19].

**Fig. 12:**
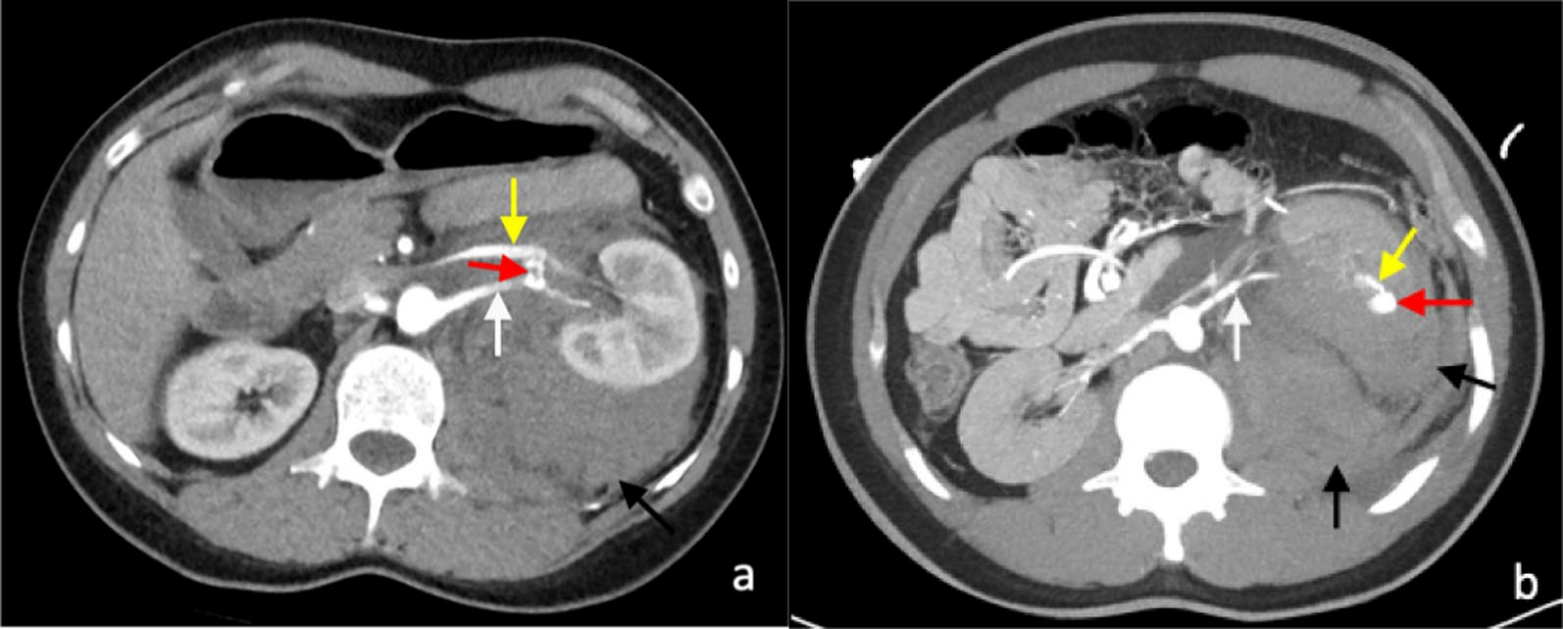
Post-contrast arterial phase axial CT images of vascular complications following non-targeted ultrasound-guided 14-gauge core biopsies of the left kidney in two patients. **a** Arteriovenous fistula. There is a fistulous connection (red arrow) between the left renal artery (white arrow) and the left renal vein (yellow) with associated early arterialised enhancement of the proximal left renal vein, medial to the arteriovenous fistula. There is an associated retroperitoneal haematoma (black arrow). **b** Arterial pseudoaneurysm. A small pseudoaneurysm (red arrow) arising from a segmental branch of the left renal artery (yellow arrow) is visualised as an adjacent small focus of rounded arterial enhancement (red arrow). The main left renal artery is also visible (white arrow). There is an associated left perinephric and retroperitoneal haematoma (black arrows)

The development of arteriovenous fistula has been reported in up to 10–15% of patients following percutaneous allograft biopsy in transplant kidney patients [61, 62], with a lower rate of up to 10% following biopsy of native kidneys [62]. Follow-up ultrasound Doppler assessment showed that the majority (> 95%) of the arteriovenous fistulae detected were asymptomatic and approximately 95% of the arteriovenous fistulae spontaneously resolved at 3 months post-biopsy [62]. A small number of the patients developed haemodynamically significant bleeding and requiring treatment. Arteriovenous fistulas lead to early arterial enhancement of the involved vein, with similar enhancement to that of the abdominal aorta and renal arteries (Fig. 12) [63].

Late complications of core biopsies of renal masses are rare, and a few cases of pseudoaneurysms and arteriovenous fistulae have been reported, following delayed presentation of the patient with haematuria, retroperitoneal haemorrhage or pain, a few months following the biopsy [60, 64, 65].

A systematic review and meta-analysis suggest that the use of smaller gauge needles may lower complication rates [66]. A randomised trial comparing the safety and diagnostic rates of renal transplant biopsy using a semiautomated biopsy gun with three differently sized biopsy needles (14, 16 or 18 gauge) found that the larger needle size had better diagnostic yield, but was associated with more post-procedural pain. The authors concluded that the use of a 16-gauge needle for renal allograft biopsies would offer the best compromise between diagnostic yield and patient acceptability [67]. In addition, patient selection may affect outcome as studies with higher serum creatinine levels, more women and higher rates of acute kidney injury recorded higher complication rates [66]. For non-targeted biopsies, polar biopsies have a lower complication rate compared with interpolar biopsies, as does avoiding the medulla and using an angle of attack of 50–70° which also increased the diagnostic yield [68].

### Ablation

Nephron-sparing procedures such as partial nephrectomy and percutaneous thermal ablation are increasingly used to treat small renal cell carcinomas (RCC), staged as T1a, i.e. not exceeding 4 cm in size, and can also be used for symptomatic control in larger T1b lesions. Percutaneous thermal ablation techniques, such as radiofrequency ablation, cryoablation, laser or microwave ablation, are also increasingly favoured, particularly in patients who are not suitable surgical candidates.

The post-procedural complications following minimally invasive ablative techniques are similar to the post-surgical complications, with the most common post-ablative complication also being haemorrhage. Most scans during or immediately following renal ablation procedures demonstrate minor perinephric haemorrhage, most commonly of no clinical significance, regardless of the ablation technique used. Haemorrhage may also be visualised along the applicator tracts following intravenous contrast administration [69]. The post-procedural haematoma may be perinephric or subcapsular. Large subcapsular haematomas can lead to renal failure due to renal parenchymal compression [70]. Injury to the collecting system or ureters can also occur following ablative therapy, leading to ureteric perforation or stenosis [69]. There are also several techniques that can be employed to protect adjacent structures including patient positioning, pneumo- or hydro-dissection, retrograde ureteral grade stent placement and irrigation and iatrogenic pneumothorax in upper pole renal masses to reduce the thermal effects [71].

Percutaneous management options of upper renal tract obstruction include percutaneous nephrostomy (PCN) or an internalised antegrade stent depending on the aetiology. PCN is often a more emergent procedure particularly in the setting of an infected, obstructed kidney. This may be due to intraluminal obstruction, for example, calculi, or extrinsic compression, in the case of retroperitoneal fibrosis. If the cause of the obstruction can be relieved and any associated infection treated, the nephrostomy can eventually be capped and subsequently removed. However, if the obstruction cannot be relieved, the placement of a ureteric stent may be necessary. This is performed percutaneously via the nephrostomy in an antegrade fashion. Other indications for PCN include urinary diversion to treat urinary leaks, fistulae and haemorrhagic cystitis, or to provide access to the urinary collecting system to deliver medication or to remove of malpositioned stents [72].

**Fig. 13:**
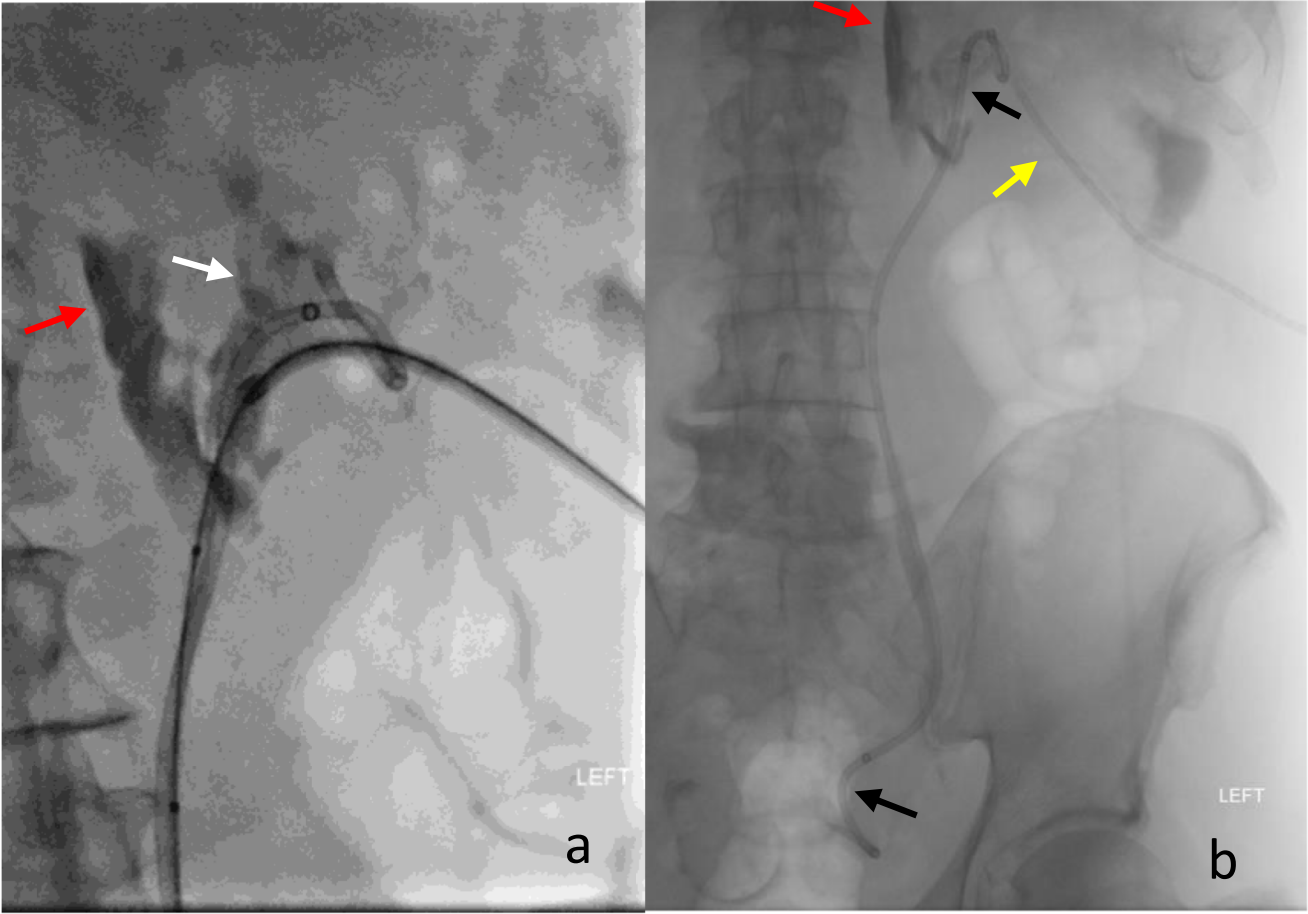
Selected AP fluoroscopic acquisitions during percutaneous nephrostomy (PCN) exchange and insertion of an antegrade stent. **a** Magnified projection demonstrating extravasation of contrast (red arrow) and opacification of the collecting system (white arrow). **b** Acquisition on completion demonstrating PCN (yellow arrow) and position of the antegrade stent (black arrows)

Major complications following percutaneous management of upper renal tract obstruction are rare, between 3 and 4%, and include bleeding requiring transfusion or surgical management or severe sepsis [73]. The overall complication rate for PCN is approximately 10% with very high insertion success rates between 82 and 100% [72]. More common minor complications include perforation of the renal pelvis, seen as contrast extravasation of contrast (Fig. 13), resulting in urine leak. With the successful placement of a PCN, this usually requires no further intervention.

## Delayed complications

### Nephrocolic fistula

**Fig. 14:**
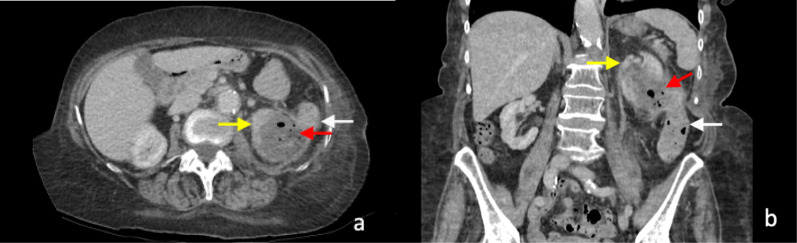
**a** Axial and **b** coronal enhanced CT images of a nephrocolonic fistula approximately 9 months following completion of 42 Gy of stereotactic ablative body radiotherapy of a left clear cell renal cell carcinoma. Faecal material is present within the fistulous connection (red arrow) between the descending colon (white arrow) and left kidney (yellow arrow)

Nephrocolic fistulas, abnormal fistulous connections between the kidney and colon, are rare. There have been a few case reports of nephrocolic fistulae following renal interventions such as lithotripsy [74–76], radiofrequency ablation [77], cryoablation [78–82] and stereotactic ablative body radiotherapy [83]. Patients presented with flank pain, recurrent urinary tract infections, pneumaturia, faecuria or haematochezia a few weeks to a few months following ablative therapy or radiotherapy. Nephrocolic fistulas can be detected by the presence of faecal material within the fistulous connection and within the affected kidney (Fig. 14).

### Tumour recurrence

**Fig. 15:**
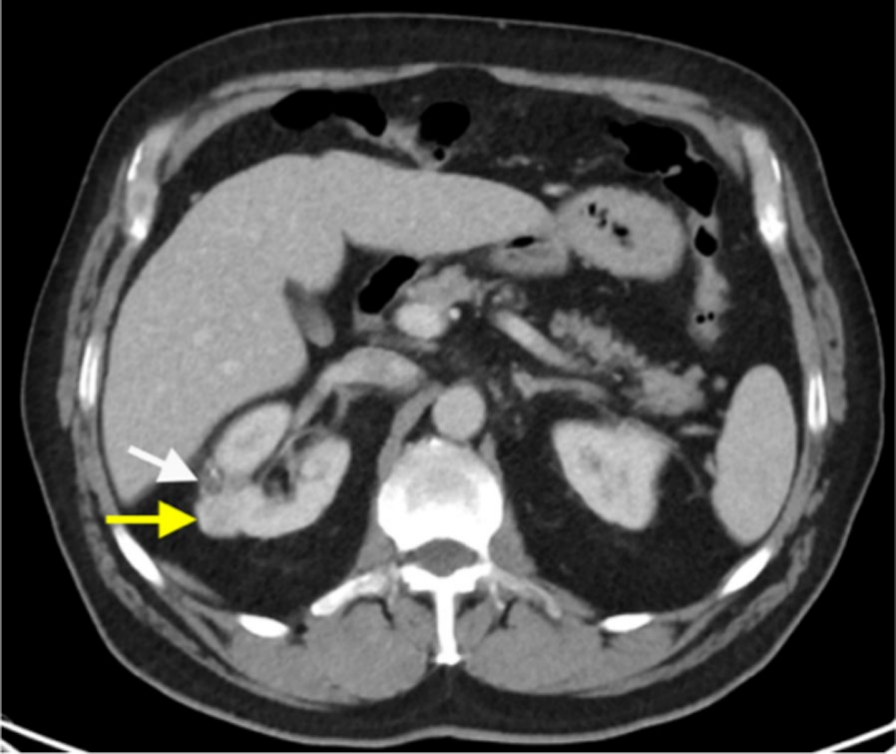
Axial contrast-enhanced CT image of recurrent renal cell carcinoma at a partial nephrectomy site at the mid-pole of the right kidney, as demonstrated by nodular enhancement (yellow arrow) at the site of the partial nephrectomy, usually hypodense (white arrow)

A small number of tumours recur following partial nephrectomy. Antic et al*.* in their literature review found that this occurred in approximately 1% of cases reviewed following partial nephrectomy with a time to recurrence ranging from approximately 3 to 24 months. Patients with underlying familial syndromes, or histologically more aggressive, or multifocal tumours may be at higher risk of developing tumour recurrence [84] (Fig. 15).

A systematic review and meta-analysis showed no significant difference in the rates of tumour recurrence following thermal ablation compared with partial nephrectomy [85]. The ablation zones appear as low-attenuation regions which may enlarge in the first few days and ultimately involute and scar. In the first few months following treatment, foci of haemorrhage may be detected as areas of increased attenuation on CT or increased signal density on MRI. A thin peripheral rim of enhancement may persist for several months following successful ablation. Successfully treated renal tumours will cease to demonstrate contrast enhancement on MRI and on CT. The presence of residual or recurrent tumour can be indicated by nodular or crescenteric contrast enhancement within the treated regions and/or by the serial increase in tumour size [4].

### Tumour seeding

**Fig. 16:**
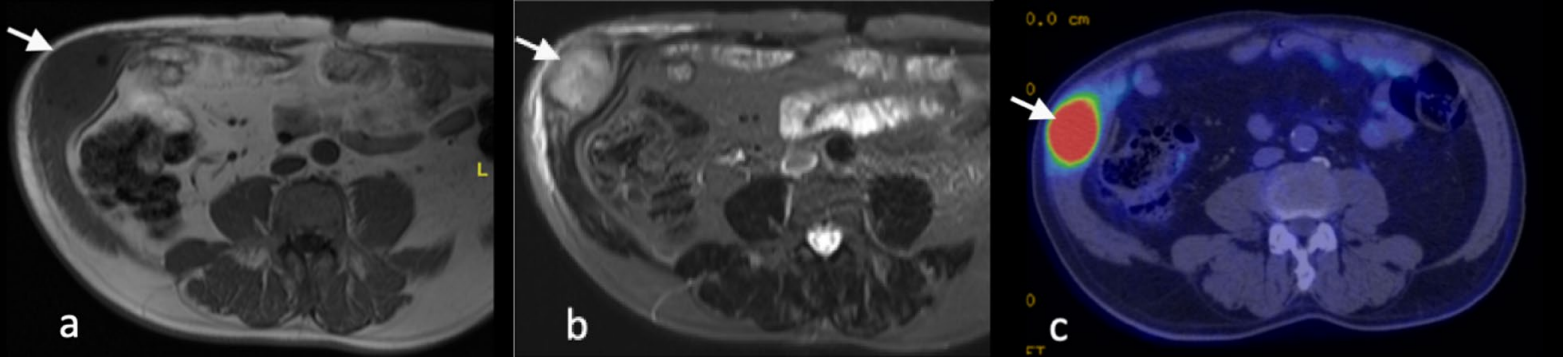
**a** T1-weighted and (**b**) T2-weighted fat suppressed MR and (**c**) PET-CT images of TCC recurrence within the right anterolateral abdominal wall scar (white arrow)

Tumour seeding along the percutaneous biopsy tract is rare, with an estimated incidence of approximately 1 in 3,000 [86]. Patients with papillary renal carcinoma [86], higher grade or stage tumour may be at higher risk of tumour seeding [87]. Tumour seeding following surgery is also extremely rare, accounting for less than 0.1% of cases [88]. Transitional cell carcinomas make up the majority of cases [89]. Two cases of seeding from renal cell carcinoma along the cryoablation probe tract have been reported [90, 91]. Intraperitoneal metastases have also been reported following radiofrequency ablation [92]. Port site metastases are associated with poor prognosis [93]. The foci of tumour seeding typically demonstrate similar imaging characteristics to the primary tumour (Fig. 16).

## Conclusions

The incidence of complications from renal interventions is expected to increase with the increasing incidence of renal pathologies. The radiologist’s role in detecting potential complications on imaging from renal interventions is, as such, more important than ever, especially with the general shift to less invasive approaches. Prompt recognition of the CT findings is vital, particularly as some of the potential complications can be life-threatening. Therefore, an understanding of early and delayed complications from a variety of renal interventions will allow the radiologist to direct prompt and appropriate management.

## Data Availability

All data generated or analysed during this study are included in this published article.

## Abbreviations

CT: Computed tomography
DSA: Digital subtraction angiography
ESWL: Extracorporeal shock wave lithotripsy
MRI: Magnetic resonance imaging
PCN: Percutaneous nephrostomy
PCNL: Percutaneous nephrolithotomy
RCC: Renal cell carcinoma
